# Neuroprotective effect of menaquinone-4 (MK-4) on transient global cerebral ischemia/reperfusion injury in rat

**DOI:** 10.1371/journal.pone.0229769

**Published:** 2020-03-09

**Authors:** Bahram Farhadi Moghadam, Masoud Fereidoni

**Affiliations:** Department of Biology, Faculty of Science, Ferdowsi University of Mashhad, Mashhad, Iran; Albany Medical College, UNITED STATES

## Abstract

Cerebral ischemia/reperfusion (I/R) injury causes cognitive deficits, excitotoxicity, neuroinflammation, oxidative stress and brain edema. Vitamin K2 (Menaquinone 4, MK-4) as a potent antioxidant can be a good candidate to ameliorate I/R consequences. This study focused on the neuroprotective effects of MK-4 for cerebral I/R insult in rat’s hippocampus. The rat model of cerebral I/R was generated by transient bilateral common carotid artery occlusion for 20 min. Rats were divided into control, I/R, I/R+DMSO (solvent (1% v/v)) and I/R+MK-4 treated (400 mg/kg, i.p.) groups. Twenty-four hours after I/R injury induction, total brain water content, superoxide dismutase (SOD) activity, nitrate/nitrite concentration and neuronal density were evaluated. In addition to quantify the apoptosis processes, TUNEL staining, as well as expression level of Bax and Bcl2, were assessed. To evaluate astrogliosis and induced neurotoxicity by I/R GFAP and GLT-1 mRNA expression level were quantified. Furthermore, pro-inflammatory cytokines including IL-1β, IL-6 and TNF-α were measured. Seven days post I/R, behavioral analysis to quantify cognitive function, as well as Nissl staining for surviving neuronal evaluation, were conducted. The findings indicated that administration of MK-4 following I/R injury improved anxiety-like behavior, short term and spatial learning and memory impairment induced by I/R. Also, MK-4 was able to diminish the increased total brain water content, apoptotic cell density, Bax/ Bcl2 ratio and GFAP mRNA expression following I/R. In addition, the high level of nitrate/nitrite, IL-6, IL-1β and TNF-α induced by I/R was reduced after MK-4 administration. However, MK-4 promotes the level of SOD activity and GLT-1 mRNA expression in I/R rat model. The findings demonstrated that MK-4 can rescue transient global cerebral I/R consequences via its anti-inflammatory and anti-oxidative stress features. MK-4 administration ameliorates neuroinflammation, neurotoxicity and neuronal cell death processes and leads to neuroprotection.

## Introduction

Transient global cerebral ischemia/reperfusion (I/R, restoration of blood flow) injury is a major consequence of cardiac arrest period and resuscitation [1]. However, short duration of cerebral ischemia (less than 10 min) can lead to neuronal death within the brain especially in the hippocampus and causes learning and memory deficits [2].

Following I/R there are three important threats for neuronal function in the brain. First excitotoxicity as a result of energy and oxygen depletion which causes the overload of calcium ions inside neurons and released glutamate excitatory neurotransmitter into the extracellular space [3, 4]. Second, oxidative and nitrosative stress in which free radicals are continuously produced as a result of oxidative phosphorylation in the mitochondria, although under physiological concentrations they serve important functions [5]. The level of free radicals is regulated by an enzymatic antioxidant like as superoxide dismutase (SOD) and non-enzymatic components including glutathione. It is shown that in the ischemic stroke the ratio between oxidants and antioxidants factors collapse which leads to oxidative stress. Neuronal cells have a high oxygen consumption and metabolic demand, therefore, they are very sensitive cell population and are more at risk for ischemic cell death. In this regard, drugs acting as free-radical scavengers or inducers of endogenous antioxidant enzymes are suitable candidates for stroke therapy [5, 6]. The third important threats following I/R for the neuronal function is neuroinflammation which plays a significant role in the pathogenesis of stroke. Tumor necrosis factor-α (TNF-α), interleukin-6 (IL-6) and interleukin-1ß (IL-1ß) are the main cytokines which initiate inflammatory reactions following brain stroke and lead to brain damages [7, 8].

Astrocytes are the most abundant and heterogeneous cell types in the central nervous system (CNS); they are involved in many protective functions including providing trophic factors, ion buffering, uptake and synthesis of excitatory neurotransmitters such as glutamate, controlling cerebral blood flow and neurogenesis in the healthy and injured brain conditions. Under pathological conditions like stroke, glial fibrillary acidic protein (GFAP) which is an intermediate filament and a selective marker of astrocytes up regulates in reactive astrocytes [9, 10]. In addition, as rapid removal of glutamate from the extracellular space is crucial for the survival and normal function of neurons, astrocytes are the cell type primarily responsible for glutamate uptake via expressing excitatory amino acid transporters (EAAT)1 and EAAT2 [11]. Among these two, EAAT2 (glutamate transporter 1, GLT-1) is the predominant subtype of glutamate transporters which is located on astrocytes of hippocampus and is responsible to maintain glutamate concentration in brain by 90% of glutamate uptake and attenuate excitotoxic cell death [12, 13]. In addition, the cell-surface protein expression of EAAT2 is regulated tightly by neuronal soluble factors and remains unaffected by exogenous glutamate levels unlike EAAT1 [14].

In the acute (minutes to hours) and late phases (hours to days) of I/R, elevation in the level of reactive oxygen species (ROS), nitrate/nitrite, cytokines and chemokines trigger immune responses and results in the activation of a variety of inflammatory cells [15]. Furthermore, it leads to intravascular accumulation of leukocytes and platelets that create an occlusion of capillaries, hypoxia and further increases in the levels of ROS and production of nitrate/nitrite (nitrogen oxide anion). In addition, degradation of extracellular matrix components by extracellular matrix metalloproteinases (MMPs) leads to blood-brain barrier (BBB) disruption which contributes to serum and blood elements release and brain edema (increased total brain water content) induction [16]. All of these factors strongly can lead to neural cells apoptosis and necrosis. Therefore, recent investigations focus on neuroprotective effects of drugs on cerebral I/R as the main tool to treatment and improvement damaged region [15, 17, 18]

Vitamin K2 (Menaquinone-4 or Menatetrenone), is one of the fat-soluble vitamin and has known as non-toxic compounds [19]. Investigations show that MK-4 has more physiological effects than K1 such as regulation of transcription factors of steroid hormones, bone metabolism, inhibition of vascular calcification and cholesterol reduction. Vitamin K is not a classical antioxidant factor, however, many studies reveal that it can potently inhibit cell death induced by oxidative stress following glutathione deficiency [20–26]. In addition, *in vitro* studies illustrated that nanomolar concentration of MK-4 can stop damages induced by oxidative stress in oligodendrocytes [6]. Moreover, Vitamin K2 has an especial feature as it can pass the BBB and reduce oxidative stress and inflammatory responses in the brain [27]. Therefore, it seems that MK-4 potentially can have neuroprotective effects on I/R. In light of potential, we investigated the effects of MK-4 administration on oxidative stress, neuroinflammation, cell death and subsequent short- and long-term learning and memory deficits induced by transient global cerebral I/R *in vivo*.

## Material and methods

### Chemicals

MK-4, Cresyl violet stain, Dimethyl sulfoxide (DMSO) were obtained from Sigma Aldrich (Germany). TUNEL kit for detection and quantification of apoptosis was purchased from Roche (Germany). Ketamine and Xylazine were purchased from Alfasan (Netherland). ELISA kits were purchased from Multi-sciences Company (China). Assay kits for SOD and NO were purchased from Navand Salamat Company (Iran).

### Animals

172 Adult male Wistar rats with weighing 250–300 gr selected at random from the animal facility of Department of Biology, Faculty of Science, Ferdowsi University of Mashhad, Mashhad, Iran. Animals were housed in standard cages under controlled room temperature (22–24°C) and humidity (45–50%) and exposed to 12:12 h light–dark cycle conditions (lights on at 08:00 am) (Table 1). The experimental procedures were approved by the National Food Chain Safety and conducted according to Institutional Animal Ethics Committee guidelines for the care and use of laboratory animals [28, 29].

**Table 1:** Number of total animals that used in the experimental groups and removed from the study.

Experimental animals: Male Wistar Rats (250-300g)	Class	Experiment	Groups	# of animals	Total number
	Behavioural studies N = 6	Open field test	Control	48 Rats	172 Rats
			I/R		
		Morris water maze test	I/R+DMSO		
			I/R+MK-4		
		Y maze test	Control		
			I/R		
			I/R+DMSO		
			I/R+MK-4		
	Histological studies N = 5	TUNNEL staining	Control	40 Rats	
			I/R		
		Nissl staining	I/R+DMSO		
			I/R+MK-4		
	Tissue analysis N = 6	Brain water content assay	Control	24 Rats	
			I/R		
			I/R+DMSO		
			I/R+MK-4		
	Biochemical studies N = 5	Nitrate/Nitrite and Superoxide dismutase assays	Control	20 Rats	
			I/R		
			I/R+DMSO		
			I/R+MK-4		
		ELISA	Control	20 Rats	
			I/R		
			I/R+DMSO		
			I/R+MK-4		
	Molecular study N = 5	Real-time PCR	Control	20 Rats	
			I/R		
			I/R+DMSO		
			I/R+MK-4		
	Removed animals	Death during and after surgery			53 Rats
		Motor activities impairment			
		Visual deficits			

### Surgical procedure for induction global cerebral I/R model

Rats were anesthetized by an intraperitoneal (i.p.) injection of Xylazine 20 mg/kg and Ketamine 100 mg/kg. The body temperature was monitored and maintained between 37.1°C and 37.3°C under free-regulating conditions with using a thermometer and heating pad during and after the surgery up to the animals recovered from anesthesia. After separation of the vagus nerve, bilateral common carotid arteries occlusion (two-vessel occlusion; 2-VO) was conducted by 20 min clamping [16, 30, 31]. After 20 min, clamps were removed for reperfusion onset either for 24 hours or for 7 days depends on different experimental sets. After surgery, animals were placed in home cages with free access to food and water. Some of the rats were removed from experiments because of: 1) death induced by ischemia, 2) movement impairment, 3) visual deficiency, 4) difficulties in reperfusion (not operated in both common carotids) and 5) seizure.

### Experimental design

**Fig 1:**
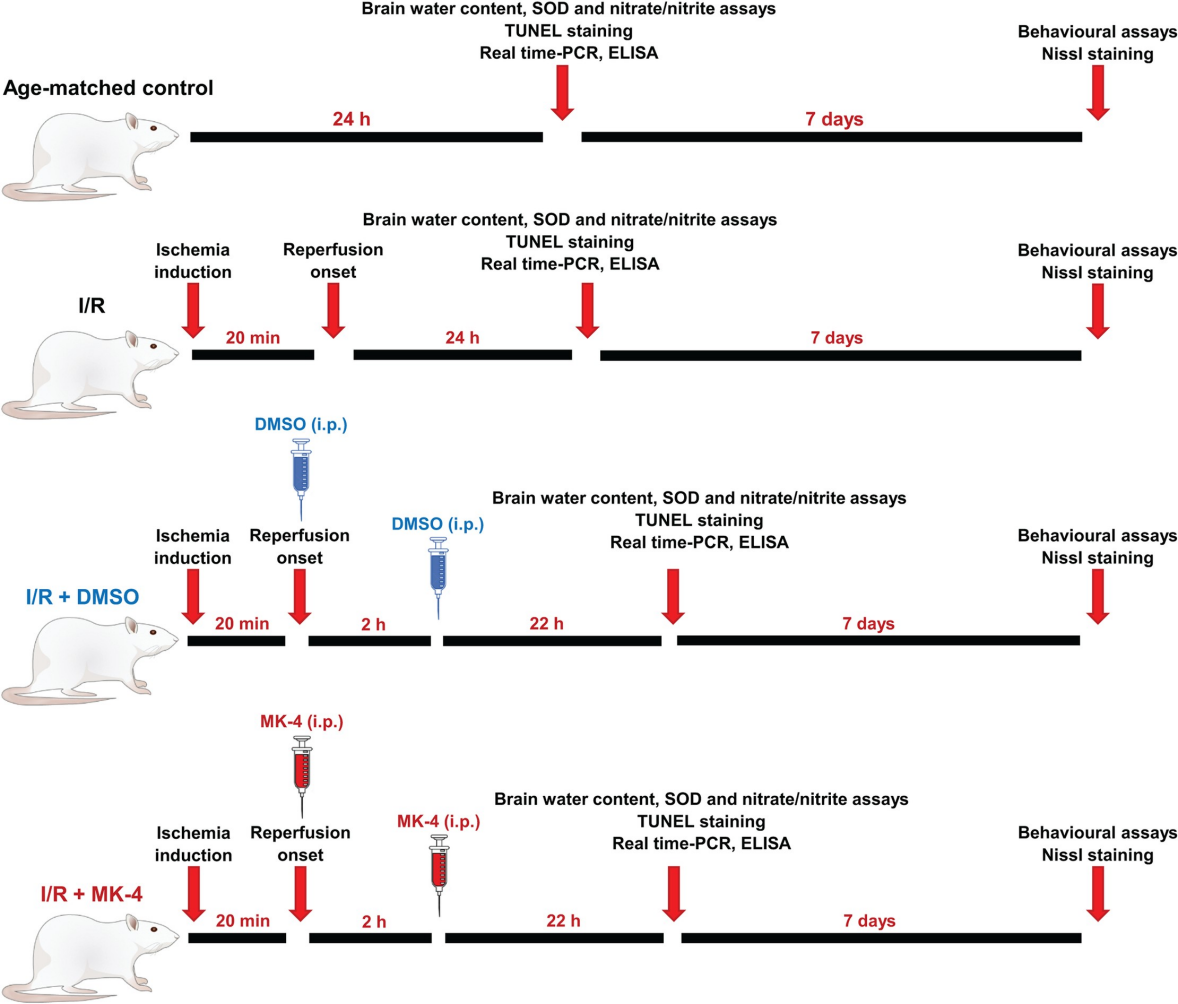
A schematic experimental timeline (N = 5–6 for each group).

216 animals were divided in 4 groups as follows: Age-matched control group, I/R injury group (sham control group), I/R injury + 2 times DMSO i.p. injection (solvent of MK-4, 1% v/v) [32], I/R injury + 400 mg/kg MK-4 (2 times 200 mg/kg i.p. injections, immediately and 2h after reperfusion). This dosage was selected empirically. At first, the dose of 100 mg/kg MK-4 i.p. was selected due to the previous investigations [33–35], however, the mortality rate of ischemic animals was high (equal I/R group). To this reason, we had to enhance the treatment dose to 400 mg/kg in two injections (200 mg/kg i.p. immediately and 200 mg/kg i.p. 2h after reperfusion). The high number of survived rats was observed subsequently. Besides, earlier studies showed that a high dose of vitamin K2 has very low toxicity [36, 37]. Behavioral tests were conducted 7 days post reperfusion in healthy animals. TUNEL and Nissl histological staining were performed 24h and 7 days after reperfusion respectively. The brain volume changes for total brain water content assessment was measured 24 hours after reperfusion. In addition, 24h after I/R injury ELISA was used for detection of pro-inflammatory cytokines including TNF-α, IL-1β, IL-6 and real-time PCR was utilized for evaluation of mRNA level of GFAP, Bax, Bcl-2 and GLT-1 (EAAT2). Then, the activity of SOD enzyme and NO concentration were assessed (Fig 1).

### Behavioral assays

For behavioral evaluation, rats were assigned to different groups (n = 6). All behavioral tests were performed at the same time of day during the light period under a dim light between 9:00 to 15:00 by a blind experimenter to all groups.

### Open field test (OFT)

The open field test was performed for general locomotor activity and anxiety-like behavior assessment. The open field arena was made of Plexiglas (72 × 72 × 36 cm), its floor divided into 25 equal areas. The rats were placed in a corner of the arena at random and allowed to explore the new environment for 5 minutes. Animal locomotion was monitored using analogue camera connected to the video tracking software ANY-Maze 5.1 [38].

### Y-Maze test

To measure short-term memory, the spontaneous alternation behavior of rats in a Y-maze was assessed 7 days after I/R. in this test the apparatus consisted of three identical arms (40 × 15 × 30 cm) positioned 120° apart and made of white Plexiglas. The animal was placed at the end of an arm and was given 8 minutes trial to move freely throughout the maze. An arm entry was considered when a rat moved all four feet into the arm. Alternation was defined as consecutive entries into each of the three arms without repetition on overlapping triplet sets. Animal movement was recorded using an analogue camera connected to the video tracking software ANY-Maze 5.1. Based on the previous study, spontaneous alternation (%) as an index for short-term memory, was calculated by the ratio of actual alternations to possible alternations, multiplied by 100 [39].

### Morris water maze (MWM)

Spatial learning and memory was assessed using the Morris water maze test. In this test, animals learn how to navigate to the hidden platform in the swimming pool using visual cues [40]. The swimming pool (150 cm diameter) was filled with water. To darken the water, the harmless black color was added to the water. The temperature of water in the swimming pool was held constant at 25 ± 1°C. The pool was surrounded by three different types of visual cues. A Plexiglas platform (10 cm diameter) was submerged 1 cm underneath the surface of the water. The location of the platform was fixed over the acquisition (NE) and reversal phases (SW). 7 days after I/R, rats were given four trials per day for five consecutive days (acquisition phase). If the animals could not find the platform within 60 s, they were guided to the hidden platform and allowed to sit on it for 15 s. A different starting point was used in each of the four trials. During the test, the time taken to reach the platform (escape latency) was recorded. For testing the reference memory, 24 h after the last days of acquisition phase, a probe trial test was performed (day 6). In the probe trial test, the platform was removed and each animal had 60 s time for free swimming. After the acquisition period, the rats were trained in a reversal learning paradigm on days 7, 8 and 9 (reversal learning phase). In this phase, the platform was located in the opposite quadrant and the procedure remained the same as acquisition training. Again 24 hours after the last reversal testing, the platform was removed and the animals were subjected to another reference memory test (day 10). To assess the ability of the animals to learn the task and check their visual ability, the visible platform task was conducted on days 11, 12 and 13 for four one-minute trials during which the platform location was changed for each trial. The platform was made visible using an aluminum foil and was elevated 3 cm above the water surface [41]. The time and distance to reach the hidden platform, time spent in different target quadrants and swimming speed were recorded using an analogue camera connected to the video tracking software ANY-Maze 5.1 [42].

### Evaluation of total brain water content

Cerebral ischemia/ reperfusion can lead to blood-brain barrier weakness and allows plasma fluid (include water and proteins) to penetrate into the intercellular space. These events form cerebral edema [43] and evaluated by total brain water content measurement [44–46].Twenty-four hours post I/R injury, rats were deeply anesthetized by Ketamine/Xylazine and sacrificed, whole brains were isolated and weighed immediately to obtain the wet weight. Then dried in an oven at 120°C for 24 h and then reweighed to record the dry weight. The liquid content of brain was calculated according to the following formula [47]:

$$Brain\;water\;content\;[\%]=\frac{Wet\;weight-Dry\;weight}{Wet\;weight}\times 100$$

### Histological experiments

Twenty-four hours after I/R, animals were deeply anesthetized by Ketamine/Xylazine, then transcardiac perfusion was performed using formalin in phosphate-buffered saline (PBS) 0.1 M, pH 7.4. Brains were isolated and after 24 h post fixation in 4% paraformaldehyde at 4 °C, the tissues were dehydrated by upgraded series of ethanol (40, 60, 70, 80, 90 and 100%, 60 min in each) and cleared in xylene, and then were embedded in paraffin. Afterward, serial coronal sections in accordance with the Paxinos atlas (between 2.8- and 4.52 mm posterior to bregma that includes hippocampal subregions CA1, CA3, dentate gyrus (DG)) with 10 μm thickness were obtained using microtome) MH2508 model, Moss Instruments Co, China) for different kinds of staining [30, 48].

### Nissl staining

In order to assess the delayed neuronal cell death using histochemical experiment, Nissl staining was done 7 days post I/R injury. In this staining, viable and nonviable cells were recognized [49]. Briefly, the sections were deparaffinized using xylene and then hydrated in downgraded series of ethanol (100, 95, 70 and 50%, 5 min in each), afterward rinsed in distilled water for 5 min. After hydration of tissues, the sections were mounted on gelatin-coated slides and were stained with 0.1% Cresyl violet for 10 min, Finally, the microscopic images were taken using a light microscope (magnification = 400X). The cell count was carried out by a person blind to the experiments. The number of viable cells were quantified in a 40000 μm^2^ area of CA1, CA3 and DG hippocampal subregions using the Image KECam software (China) [50–52].Seven sections from each brain sample with 100 μm interval (select one section from 10 section) were prepared and 4 regions of interest (ROI) were selected in each area randomly in which cells were counted.

### TUNEL staining

Terminal deoxynucleotidyl transferase dUTP nick end labeling (TUNEL) is a method for detecting DNA fragmentation by labeling the 3′-hydroxyl termini in the double-strand DNA breaks generated during apoptosis. However, still, it is not possible to distinguish apoptotic or necrotic cell death using TUNEL staining [53]. Usually for evaluation of apoptosis after stroke TUNEL staining uses in cerebral ischemia. Previous studies showed that following I/R, TUNEL-positive cells can be found from 1 hour after occlusion and reach their maximum density after 24 hours [54]. In this study, TUNEL staining was performed according to the instructions (*in Situ* Cell Death Detection Kit, Roche, Germany). Briefly, sections were deparaffinized by xylene, ethanol and PBS, then permeabilized by permeabilization solution (Triton-X, Trisodium citrate) 8 min at 4 °C. Afterward, sections were washed with PBS, then Proteinase-K was added for 8 min at room temperature, and then sections were washed with PBS again. After removal of equilibration buffer, 20–30 μl of rTdT was added with coverslips and incubated in a dark moisture chamber at 37°C for 60 min. Negative and positive control slides were treated with DNaseI. Finally, after incubation, samples were washed by PBS [55]. Four sections from each brain sample with 200 μm interval (select one section from 20 section) were prepared and 4 regions of interest (ROI) were selected in each area randomly in which cells were counted. The slides were imaged by a fluorescent microscope (magnification = 400X). Apoptotic neurons in CA1, CA3 and DG hippocampal subregions were counted in a 36000 μm^2^ area and were analyzed using Image KECam software. The cell count was carried out by a person blind to the experiments.

### Nitrate/nitrite (Nitric oxide anions) assessment

Nitric oxide level in the homogenized hippocampal tissue was assessed using Griess assay that measures nitrite levels. In this analytical chemistry method, using a colorimetric reaction the NO_2_-(nitrite) level in aqua solution was measured [56]. This method is a reaction based on the synthesis of the “Diazobenzolamidonaphtol” (azo dye). To this reason under acidic conditions nitrite of tissue sample reacts with the amino group of sulfanilic acid to create the diazonium cation, which couples to -naphthylamine to form the azo dye (red–violet color) [56]. Here, Griess assay was performed using Natrix kit (Navand Salamat, Iran) due to the manufacturer’s manual. Briefly, the hippocampus was isolated and homogenized in PBS, then centrifuged in 140000 g with supernatant being collected. Supernatant samples and standard solution were poured on 96-well plates with 3 times repetition. Then the light absorbance was assessed at 570 nm by Elisa reader apparatus (Stat Fax 2100 Microplate Reader). Finally, the nitrate/nitrite concentration due to the weight of hippocampus and standard nitrite curve was analyzed.

### Superoxide dismutase (SOD) enzyme activity assay

SOD is an enzyme which catalyzes the superoxide (O_2_^−^) radical into oxygen (O_2_) or hydrogen peroxide (H_2_O_2_). SOD is an important antioxidant in almost all cell types which faced oxygen [57]. To assess SOD activity, we used Nasdox kit (Navand Salamat, Iran) that is based on the inhibition of Pyrogallol autoxidation by SOD activity. The assay system contained Pentetic acid, catalase, Tris-Cacodylate buffer at pH 8.5.

After isolation of hippocampi and homogenization in PBS, supernatant was collected following centrifugation. The assay solution was mixed with supernatant and after addition of Pyrogallol solution, the assay mixture was transferred to a 1.5 ml cuvette and the rate of increase in the absorbance at 420 nm was recorded from 0 to 3 min (every 1 min) using spectrophotometer. After addition of pyrogallol, the increase of absorbance at 420 nm was inhibited in the presence of SOD. One unit of SOD is described as the amount of enzyme required to cause 50% inhibition of pyrogallol autoxidation in the supernatant mixture. Results were expressed in units per mg protein for tissue homogenate. Finally, the difference between optical density (OD) of samples and control was calculated as ΔOD and used in the following formula for calculation of SOD activity [58–60]:

$$SOD\;activity\;\left(\frac{U}{ml}or\;mg\;protein\right)=\frac{{\Delta OD}_{Test}}{{\Delta OD}_{Control}}\times 100$$

### Real time PCR

To measure gene expression, RNA was extracted by conventional TRIzol method (RNX-Plus, SinaClone, Iran). The concentration and integrity of extracted RNA was determined by UV spectrophotometry and gel electrophoresis. cDNA was synthesized using Revert Aid First Strand cDNA Synthesis Kit (Thermo Fisher, USA). The primers were designed and synthesized by Macrogen, Inc (Seoul, South Korea). In this study, *GLT-1*, *GFAP*, *Bax* (pro-apoptotic) and *Bcl-2* (anti-apoptotic) genes were chosen as targets. The glyceraldehyde-3-phosphate dehydrogenase (*GAPDH*) gene was also selected as a housekeeping gene. Primers were designed by the primer-Blast system (Table 2). The reaction system was 2X SYBR Green PCR Master mix (Parstous, Iran) 12.5 μl + upstream and downstream primers (10 pmol/ul) 1 μl each + cDNA template 1 μl, adding water to the total volume of 25 ul. The reaction condition was the same for all genes analyzed: initial denaturation at 95°C for 2 min, and 40 cycles of 95°C for 15 sec, 58°C for 20 sec, 72°C for 25 sec. Amplification curves were constructed, and the relative expression of mRNA was calculated by 2-ΔΔCq method [61, 62].

**Table 2:** List of primer sequences used for RT-PCR analysis. F: Forward primer, R: reverse primer, Tm: Melting temperature.

Genes	Tm (°C)	Primer sequences
**Bax**	**F: 60.29** **R: 60.32**	**F: TTGCTACAGGGTTTCATCCAGG** **R: CACTCGCTCAGCTTCTTGGT**
**Bcl-2**	**F: 59.89** **R: 60.55**	**F: CTTTGAGTTCGGTGGGGTCA** **R: AGTTCCACAAAGGCATCCCAG**
**GFAP**	**F: 59.83** **R: 60.45**	**F: GAGTTACCAGGAGGCACTCG** **R: GGTGATGCGGTTTTCTTCGC**
**GLT-1**	**F: 60.33** **R: 59.97**	**F: TGGACTGGCTGCTGGATAGA** **R: GCTCGGACTTGGAAAGGTGA**
**GAPDH**	**F: 60.68** **R: 60.39**	**F: AGTGCCAGCCTCGTCTCATA** **R: ATGAAGGGGTCGTTGATGGC**

### Enzyme-linked immunosorbent assay (ELISA)

The levels of pro-inflammatory cytokines IL-1β, IL6 and TNF-α in the hippocampus was measured using ELISA method. For this purpose, hippocampi were isolated and homogenized in cell lysis buffer (5 mg of tissue per 500μl of lysis buffer), then centrifuged at 10000 rpm at 4°C for 15 min. The Supernatant was collected into the new tube and diluted 5 times with diluents buffer. Then 100 μl of samples were added to each well of the 96 well plates of ELISA kits which was coated with respective antibodies. After incubation, the optical density was measured using an ELISA reader at 570 nm wavelength. Sandwich ELISA was performed according to the manufacturer's protocol (IL-1B, IL6 and TNF-α rat ELISA kits (MultiScience (Lianke) Biotech CO., Ltd) [63].

### Statistical analysis

Data were evaluated and plotted by GraphPad Prism version 7 (GraphPad Software, Inc. USA) and expressed as mean±SEM. Two-way repeated measure ANOVA was applied for MWM experiment data analysis, whereas one-way ANOVA was used in other experiments. Tukey correction for multiple comparisons was used as a post hoc test. The minimum significance value was considered as p < 0.05. All experiments were analyzed in a blind fashion.

## Results

### Positive effect of 400 mg/kg MK-4 administration on mortality rate in I/R rat model

**Fig 2:**
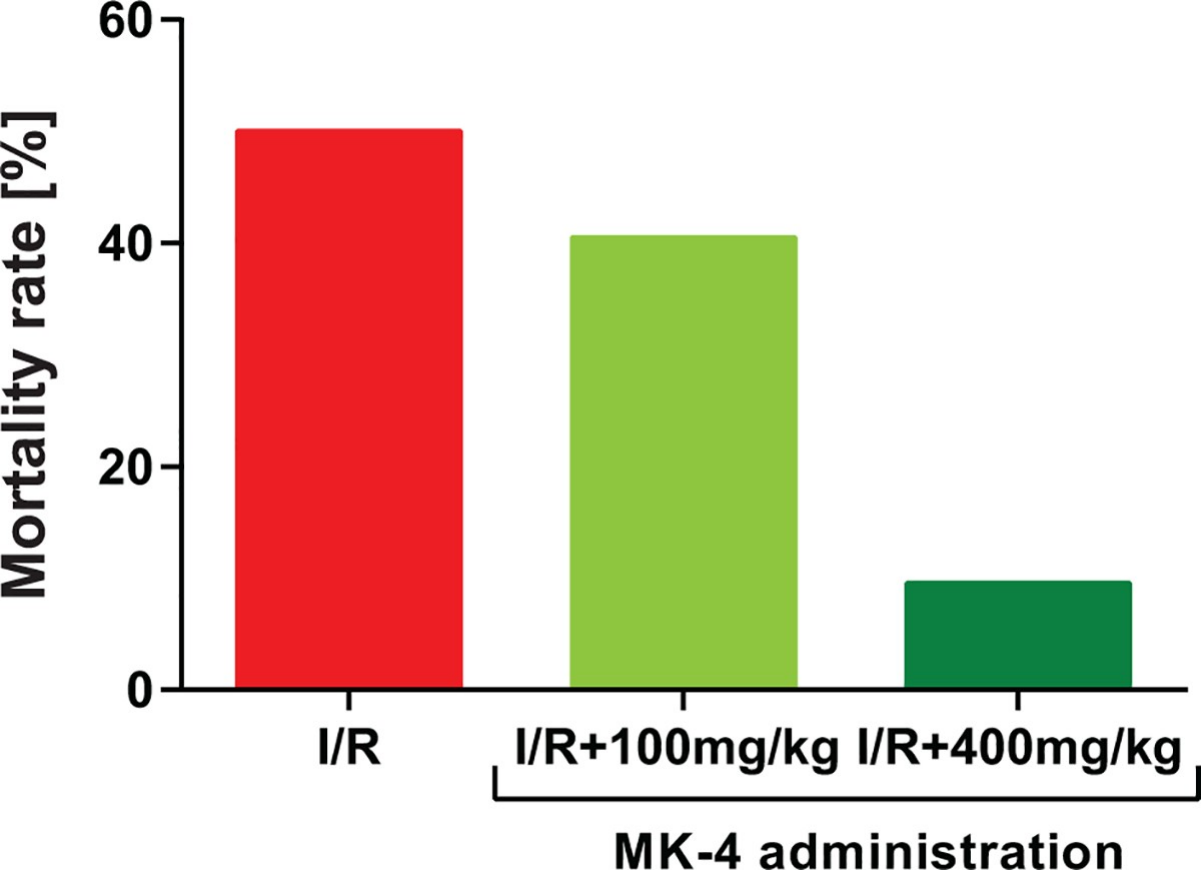
Positive effect of 400 mg/kg MK-4 administration on mortality rate in I/R rat model. Although administration of 100 mg/kg MK-4 following I/R showed a similar mortality rate compared to I/R group. The dose of 400 mg/kg MK-4 in two injections (200 mg/kg i.p. immediately and 200 mg/kg i.p. 2h after reperfusion) led to reduced mortality rate compared to I/R group.

In order to empirically selection of the neuroprotective administration dose of MK-4 for I/R injury, first, the dose of 100 mg/kg MK-4 i.p. was selected due to the previous investigations [33–35]. The findings indicated that the mortality rate of ischemic animals was high (equal to I/R group). For this reason, the treatment dose to 400 mg/kg in two injections (200 mg/kg i.p. immediately and 200 mg/kg i.p. 2h after reperfusion) was increased and the high number of survived rats was observed subsequently (Fig 2). Therefore, the treatment dose of 400 mg/kg (in two injections) was selected in this study. In line with our findings, previous investigations revealed that a high dose of vitamin K2 has very low toxicity [36, 37].

### Positive effect of MK-4 administration on behavioral deficits in I/R rat model

**Fig 3:**
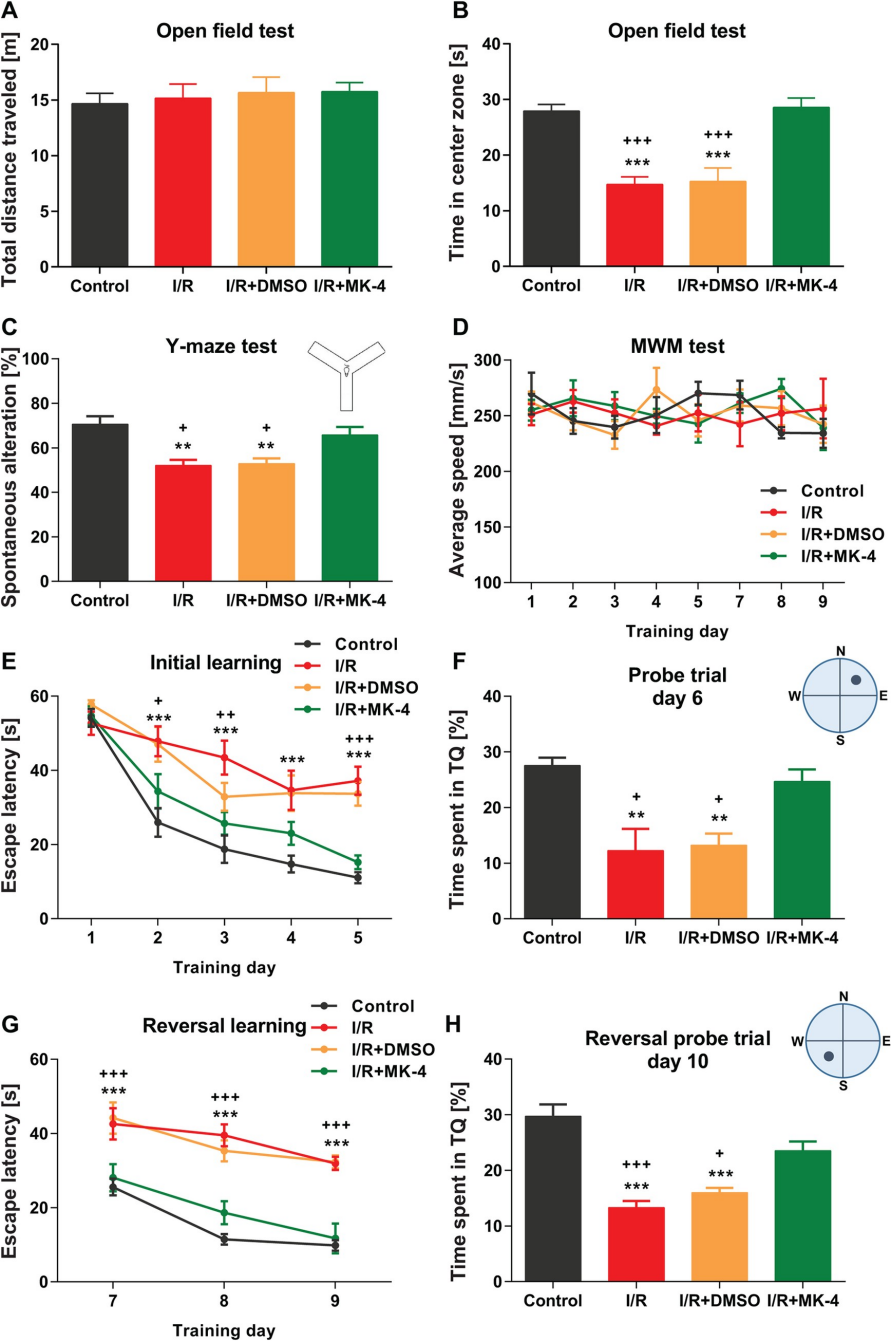
The positive effects of MK-4 administration on behavioral deficits induced by I/R injury. **(A)** In the open field test, total distance traveled was not changed significantly following I/R injury induction. **(B)** Time spent in the center zone of open field arena was reduced post I/R, however, i.p. administration of 400 mg/kg MK-4 (200 mg/kg immediately and 2 h after I/R injury) led to compensate the phenotype. **(C)** Spontaneous alternation behavior in the Y maze test was reduced in rat I/R model, but it backed to the control level following MK-4 administration in I/R animals. **(D)** In the Morris water maze test, swimming speed was not altered significantly between groups. **(E-H)** Escape latency during the initial **(E-F)** and reversal phases **(G-H)** of Morris water maze was higher in I/R animals. In addition, I/R rats spent less time in target quadrants (TQ) in both reference memory tests compared to control. Following MK-4 administration in I/R animals, the phenotypes disappeared and the results were comparable to control group. Data are presented as mean±SEM. ** p <0.01 and *** p < 0.001 compared to control, + p < 0.05, ++ p < 0.001 and +++ p < 0.001 compared to I/R+MK4 group (n = 6).

In order to evaluate the neuroprotective role of MK-4 administration for I/R injury induced brain dysfunction, behavioral experiments were conducted. First, general locomotor activity and anxiety-like behavior of animals in different experimental groups were analyzed using the open-field test (Fig 3A and 3B). The results revealed that neither control nor other experimental groups showed any deficits in locomotor activity, as total distance traveled were indistinguishable between all tested groups (Fig 3A). However, rats in I/R (14.71 s) and I/R + DMSO (15.24 s) groups spent significantly less time in the center zone of open field arena compared to control (27.91 s) group (p < 0.001). But the time spent in the center zone was significantly increased in I/R animals which received MK-4 (28.56 s), as the phenotype in this group was comparable with control group (one-way ANOVA: F_OFT_ (3, 16) = 19.40, p < 0.001, Fig 3B). This result indicated that I/R induces anxiety-like behavior, however, administration of MK-4 can reverse the phenotype.

To assess the spatial working/short term memory in I/R rat model following MK-4 application, spontaneous alternation was evaluated in Y-maze test (Fig 3C). The result showed that I/R (52.07%) injury induction alone or accompanied with DMSO (52.94%) injection led to diminished percentage of spontaneous alternation compared to control (70.59%) group (p < 0.01), however, the phenotype in I/R animals backed to control level following MK-4 (65.76%) administration (one-way ANOVA: F_Y-maze_ (3, 24) = 8.93, p = 0.0004, Fig 3C). Thus, cerebral I/R injury impaired working memory but MK-4 application can improve this deficit.

Previously, it was shown that cerebral I/R injury impaired spatial learning and memory in ischemic rats [64]. Here, also the spatial learning and memory in initial and reversal phases of MWM test in different experimental groups (control, I/R, I/R + DMSO and I/R + MK-4) were assessed. At initial and reversal acquisition phases, swim speed was not altered significantly in all tested groups (two-way RM ANOVA: F_Swim speed_ (3, 20) = 0.20, p = 0.89, Fig 3D). During 5 days of initial learning, the escape latency reduced in all tested animals, however, swimming time to reach the hidden platform was significantly higher in I/R and I/R + DMSO groups compared to control animals (p < 0.001). Following MK-4 administration in I/R animals, escape latency was reduced significantly compared I/R rats (p < 0.05) and it did not show any significant difference in comparison with control group (two-way RM ANOVA: F_Escape latency_ (3, 20) = 16,36, p < 0.001, Fig 3E). Analysis of memory retrieval on day 6 following initial learning showed that I/R (12.25%) and I/R + DMSO (13.23%) animals spent less time in the target quadrant (NE) than control (27.55%) rats (p < 0.01). But MK-4 (24.66%) injection following I/R induction led to elevate time spent in target quadrant compared to I/R animals (p < 0.05) (Fig 3F). In the reversal phase of the Morris water maze test, the platform was moved to the opposite quadrant (SW) and the rats were trained for another 3 consecutive days. This test needs cognitive flexibility [65]. The results indicated that escape latency was significantly higher in I/R and I/R + DMSO tested groups compared to control, in addition here injection of MK-4 reversed the phenotype in I/R animals (two-way RM ANOVA: F_Escape latency_ (3, 20) = 22,58, p < 0.001, Fig 3G). On the 10th day, the reversal reference memory test was performed. No differences between I/R + MK-4 (23.50%) and control (29.72%) groups was observed however I/R (13.28%) and I/R + DMSO (15.98%) rats spent less time in target quadrants compared to control (Fig 3H).

At the end of water maze sessions, the mice were trained for another 3 consecutive days with a visible platform. The escape latency did not show any significant differences between all tested groups which represent all of the animals have intact power sight and phenotypes observed in the water maze test would be purely referable to cognitive function impairment (Data are not shown). Overall, these data revealed that cerebral I/R injury led to cognitive deficits, however, MK-4 injection following I/R improved this impairment.

### Reduction of total brain water content induced by I/R injury following MK-4 administration in I/R rat model

**Fig 4:**
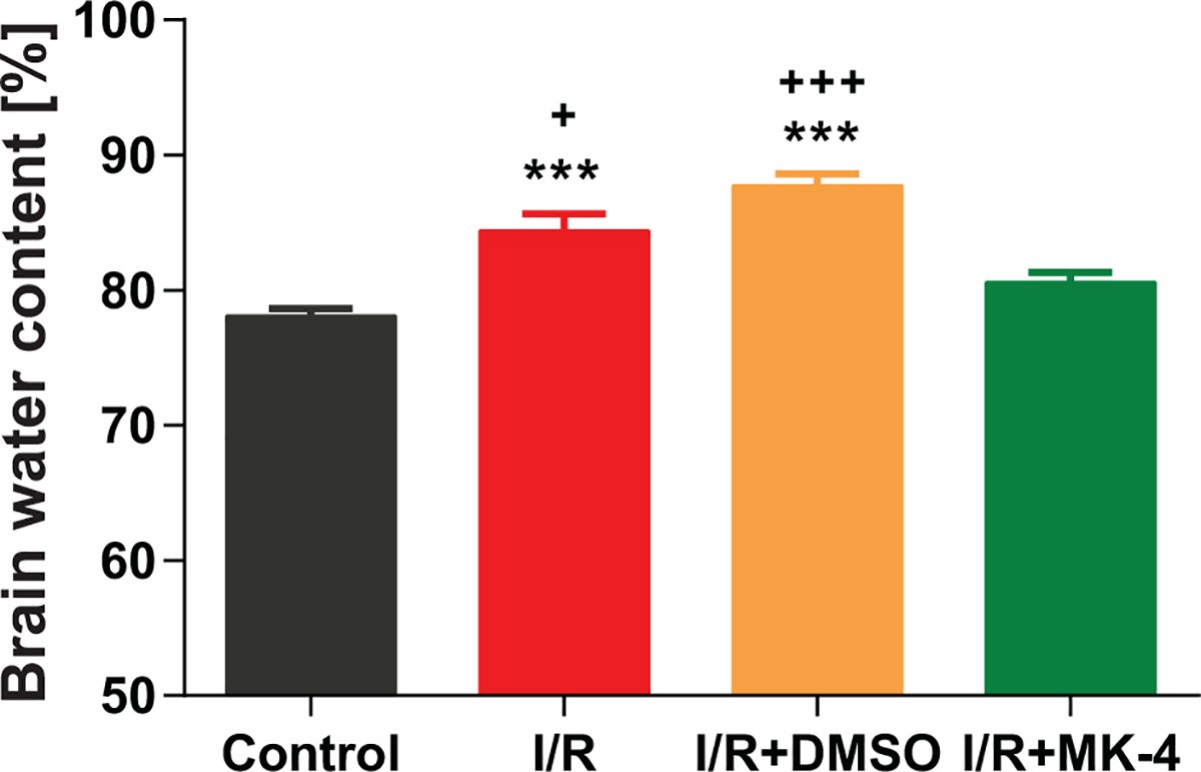
Reduction of brain water content following MK-4 application in I/R rat model. Twenty-four h after I/R injury, brain water content as an index of brain edema was increased. But i.p. MK-4 injection could decrease total brain water content considerably. Data are presented as mean±SEM. *** p < 0.001 compared to control, + p < 0.05 and +++ p < 0.001 compared to I/R+MK4 group (n = 6).

Total cerebral water content is an important contributor to poor brain functional outcome following I/R injury. Here, the finding of brain edema assessment showed that brain water content percentage was significantly higher in I/R (84.37%) and I/R + DMSO (87.72%) groups compared to control (78.10%) (p < 0.001, Fig 4). However, following MK-4 (80.56%) administration in I/R rats, brain water content was comparable to control group and was less than I/R and I/R + DMSO tested animals (p < 0.05) (Fig 4). It is important to know that even small alterations in the brain water content can reflect the huge changes in the absolute water content of the brain [47]. Therefore, it can lead to intracranial pressure and blood flow impairment and cause brain function deficits.

### Reduction of neuronal cell death induced by I/R injury following MK-4 administration in I/R rat model

Previous findings demonstrated that different forms of cell death such as apoptosis and necrosis elevate following cerebral ischemia injury. In addition, hippocampal neurons, especially in CA1 hippocampal subregion, easily can be damaged during cerebral ischemia [66].

**Fig 5:**
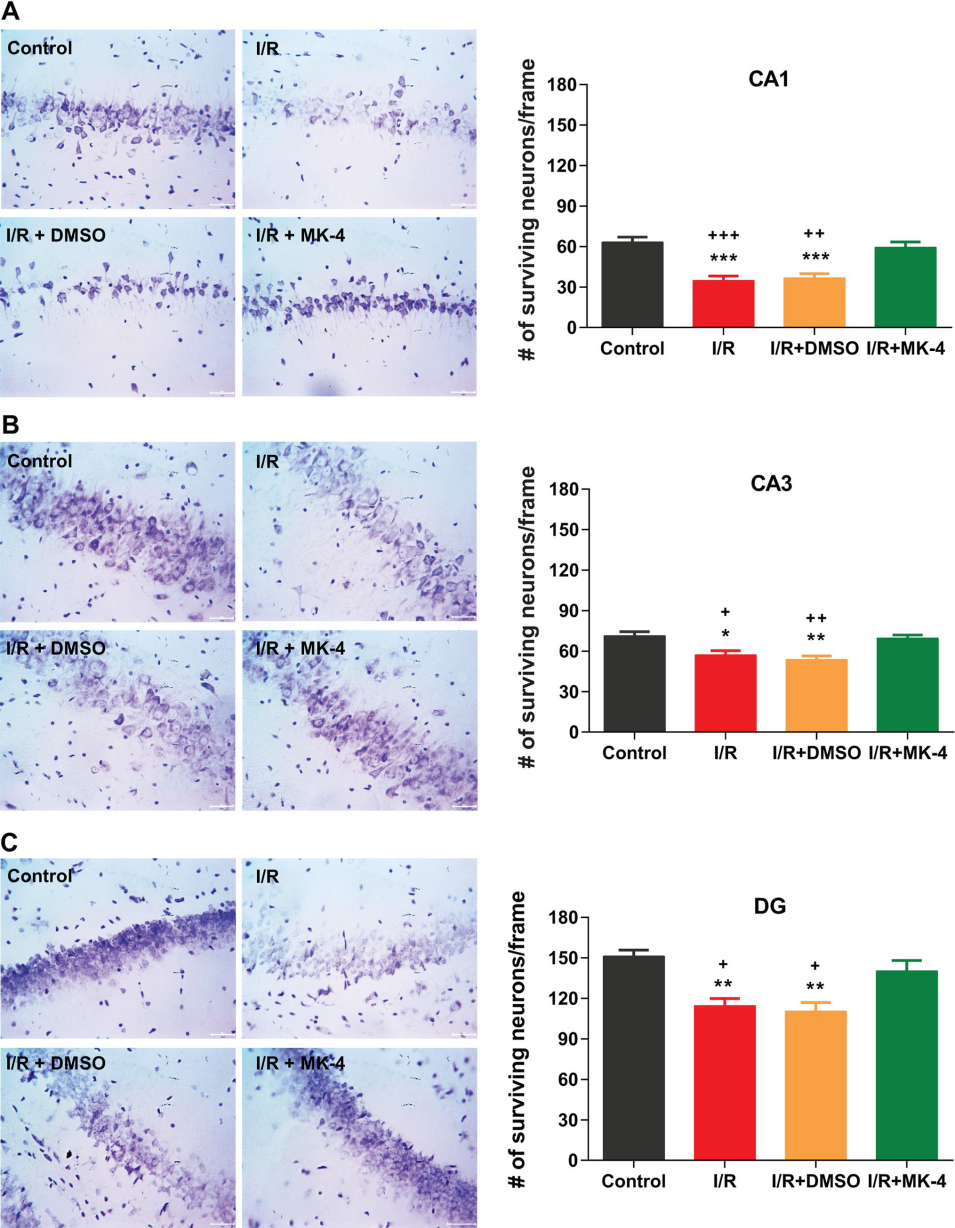
MK-4 administration attenuated neuronal loss in the hippocampal subregions induced by I/R injury. Using Nissl staining neuronal density was quantified in different hippocampal subregions. 7 days after reperfusion neuronal density was diminished in **(A)** CA1, **(B)** CA3 and **(C)** DG hippocampal subregions. But MK-4 injection could prevent neuronal loss induced by I/R injury. (Scale bar = 20 μm in representative examples of Nissl staining). Data are presented as mean±SEM. * p < 0.05, ** p < 0.01 and *** p < 0.001 compared to control, + p < 0.05, ++ p < 0.01 and +++ p < 0.001 compared to I/R+MK4 group (n = 6).

Here, also as the results of Nissl staining revealed, in control group, neuronal cells in different CA1 (Fig 5A), CA3 (Fig 5B) and DG (Fig 5C) hippocampal subregions showed clear nuclei and nucleoli, while in I/R and I/R + DMSO groups following ischemia, a significant neuronal damage was observed compared to control group (Fig 5). Moreover, in different hippocampal subregions, neuronal cells in I/R group indicated significant karyopyknotic nuclei which refer to an irreversible condensation of chromatin in the nucleus of a cell following necrosis or apoptosis [67], compared to control (Fig 5). Furthermore, the density of surviving neuronal cells was diminished in I/R and I/R + DMSO groups compared to control (p < 0.05), but interestingly administration of MK-4 following I/R injury led to an increase in the number of surviving cells in different hippocampal subregions (p < 0.05) compared to I/R and I/R + DMSO groups (Fig 5).

### Reduction of apoptotic cells population induced by I/R injury following MK-4 administration in I/R rat model

**Fig 6:**
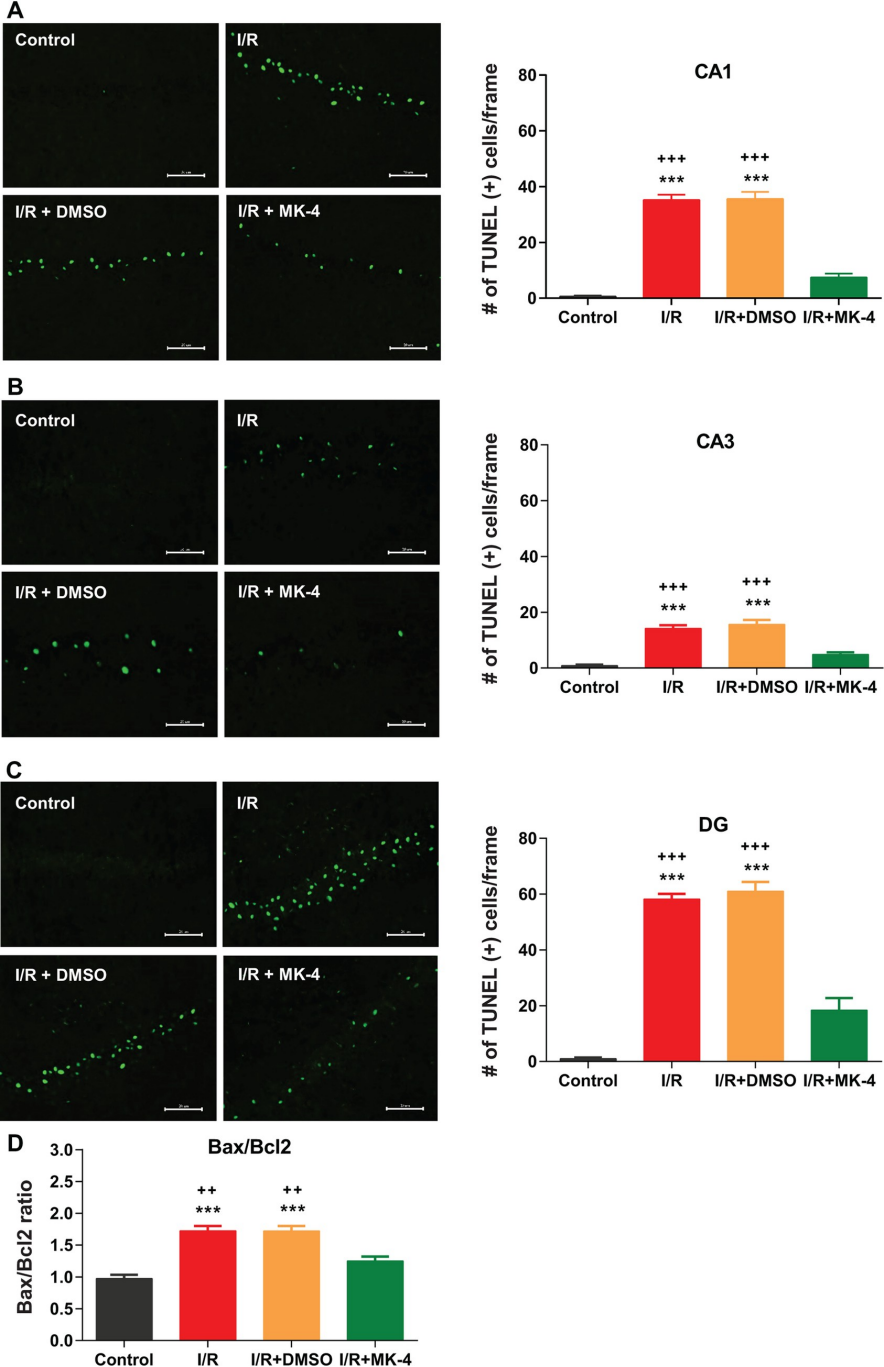
MK-4 application prevented apoptosis induction by I/R injury. Using TUNEL staining, apoptotic cells were labeled in different hippocampal subregions. 24 h after reperfusion the number of TUNEL positive cells was increased in **(A)** CA1, **(B)** CA3 and **(C)** DG hippocampal subregions. However, MK-4 administration led to a reduction in apoptotic cell density induced by I/R injury. (Scale bar = 20 μm in representative examples of TUNEL staining). **(D)** The mRNA expression level of Bax and Bcl-2 genes was quantified using real-time PCR relative to GAPDH reference gene in rat’s hippocampus. I/R injury elevated Bax/Bcl-2 ratio in the hippocampus compared to control, but MK-4 administration significantly modulates the I/R-induced increase in Bax/Bcl-2 ratio. Data are presented as mean±SEM. *** p < 0.001 compared to control, ++ p < 0.01 and +++ p < 0.001 compared to I/R+MK4 group (n = 6).

Neuronal cell apoptosis occurs following cerebral I/R as a consequence of the expression of apoptosis-related proteins such as Bcl-2 and Bax [68]. Here first TUNEL staining was used for labeling DNA fragmentation in the nucleus of apoptotic dead cells [53]. The results of TUNEL staining showed that 24 hours after I/R injury apoptotic cells were appeared in the hippocampus (Fig 6). In control group, TUNEL-stained cells with dark nuclei and reduced cytoplasm rarely were detected in different hippocampal CA1 (Fig 6A), CA3 (Fig 6B) and DG (Fig 6C) subregions. Whereas in I/R and I/R + DMSO groups, the density of TUNEL-positive cells was increased in hippocampal subregions compared to control (p < 0.001). Administration of MK-4 following I/R injury led to significantly diminished apoptotic cell density (p < 0.001) in the CA1, CA3 and DG hippocampal subregions compared to I/R and I/R + DMSO groups (Fig 6). In addition, using real-time PCR, mRNA expression level of Bax and Bcl-2 in the hippocampus of the rats in each experimental groups were quantified (Fig 6D). Bcl-2 is the apoptosis inhibitory protein, however, Bax is the apoptosis-promoting protein. The Bax/Bcl-2 ratio is also considered as a marker for apoptosis induction by I/R injury. When the Bax/Bcl-2 ratio decreases, cell apoptosis is inhibited and when this ratio increases, apoptosis is promoted [68]. Here the result of Bax/Bcl-2 ratio evaluation indicated that in the hippocampus of I/R and I/R + DMSO rats the ratio of Bax/Bcl-2 increased compared to control (p < 0.001). While MK-4 injection following I/R injury could decrease this ratio in comparison with I/R and I/R + DMSO animals (p < 0.01) and backed to control level (Fig 6D).

### Reduction of oxidative stress induced by I/R injury following MK-4 administration in I/R rat model

**Fig 7:**
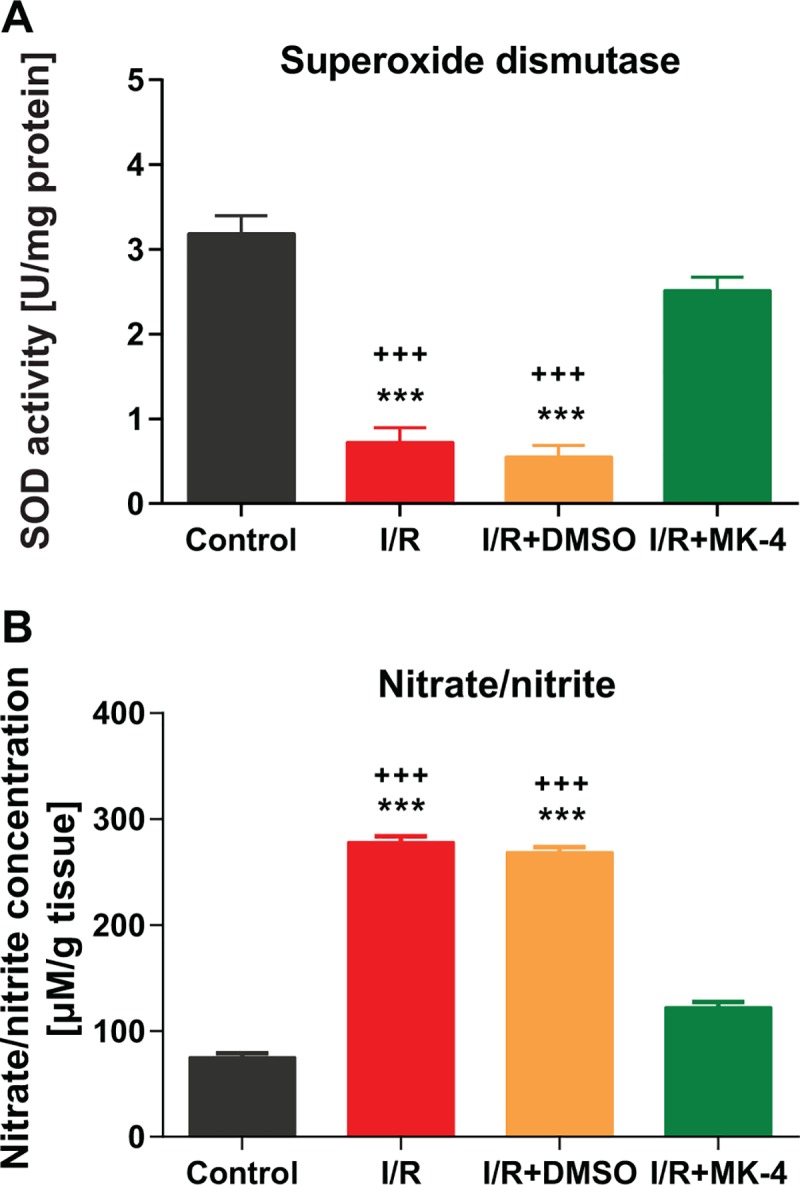
Diminished oxidative stress induced by I/R injury following MK-4 administration in I/R rat model. **(A)** SOD activity in the hippocampus was reduced following I/R injury, however, this phenotype was backed to the control level after MK-4 injection in I/R rat model. **(B)** Nitrate/nitrite level increased significantly following I/R injury but MK-4 administration prevented it. Data are presented as mean±SEM. *** p < 0.001 compared to control and +++ p < 0.001 compared to I/R+MK4 group (n = 6).

Oxidative stress is considered as a recognized factor in the initiation of I/R injury. I/R injury induces mitochondrial homeostasis dysregulation which leads to substantial oxygen and nitrogen species (RONS) release [69]. SOD is an enzyme which catalyzes the superoxide (O_2_^−^) radical into oxygen (O_2_) or hydrogen peroxide (H_2_O_2_). Oxygen metabolism in the living cells can lead to superoxide production and, if not regulated, causes many types of cell damage. Therefore, SOD is a crucial antioxidant defense in most of all living cells exposed to oxygen especially following reperfusion [57]. In this study it was shown that SOD activity was reduced in I/R and I/R + DMSO groups compared to control (p <0.001), however, MK-4 injection as a potential antioxidant led to elevation of SOD activity in the hippocampus of ischemic rats compared I/R and I/R + DMSO animals (p < 0.001) (Fig 7A). Moreover, previously it has been shown that immediately after ischemia, the concentration of nitric oxide anions (nitrate/nitrite) arise in the brain and it can induce relevant brain damage [70]. Here the result of nitrate/nitrite assessment revealed that NO concentration significantly increased in the hippocampal tissue in I/R and I/R + DMSO groups compared to control (p < 0.001). However, MK-4 administration following I/R injury could decrease the amount of NO production in comparison with I/R and I/R + DMSO animals (p < 0.001) (Fig 7B).

### Induction of EAAT2 (glutamate transporter 1, GLT-1) following MK-4 administration in I/R rat model

**Fig 8:**
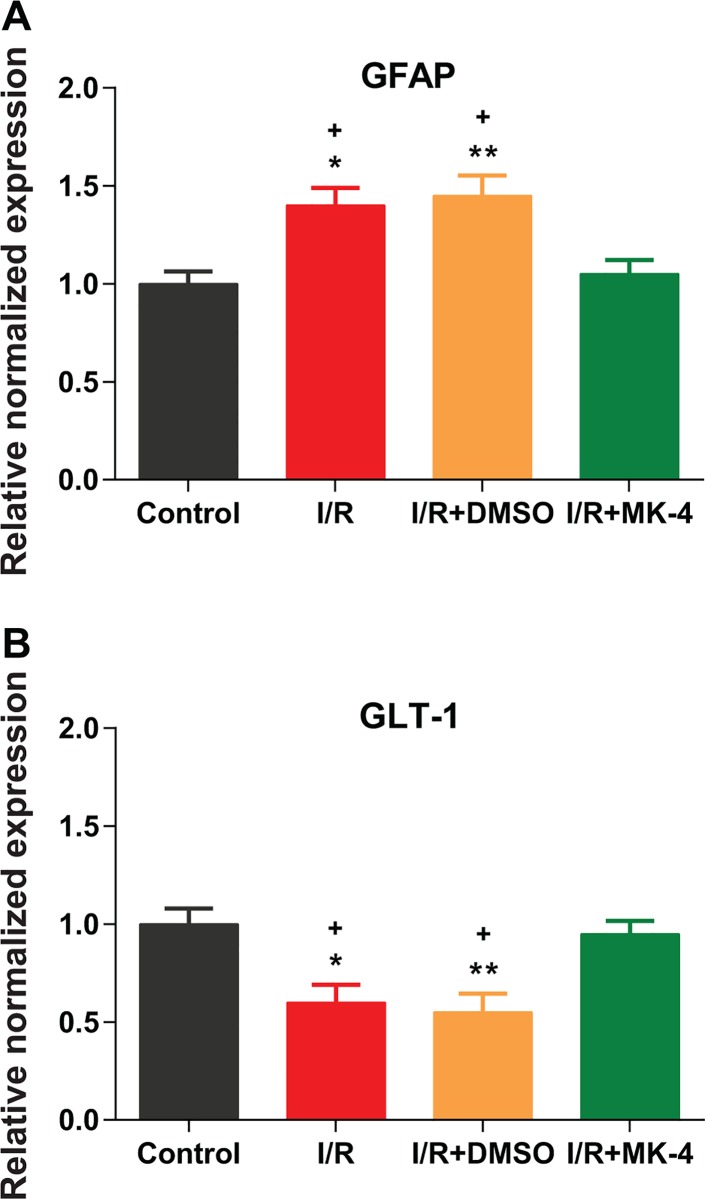
Reduced astrogliosis and EAAT2 (glutamate transporter 1, GLT-1) induction following MK-4 administration in I/R rat model. The mRNA expression level of GFAP and GLT-1 genes was quantified using real-time PCR relative to GAPDH reference gene in rat’s hippocampus. **(A)** I/R injury induced astrogliosis by elevated GFAP expression level, but MK-4 injection inhibited it. **(B)** GLT-1 mRNA level was reduced following I/R injury and MK-4 administration backed it to the control level. Data are presented as mean±SEM. * p < 0.05 and ** p < 0.01 compared to control, + p < 0.05 compared to I/R+MK4 group (n = 6).

Astrocytes—the most abundant cell type in the CNS—play an important role in many pathological conditions of the CNS, especially ischemic stroke. Reactive astrogliosis and glial scar formation is one of the relevant pathological characterizations of I/R injury. The elevation in the GFAP expression is a marker for reactive astrogliosis [71]. Here, as results of real-time PCR showed mRNA expression of GFAP was significantly increased in I/R and I/R + DMSO animals (p < 0.05) compared to control (Fig 8A). However, MK-4 application post I/R injury led to GFAP expression reduction compared to I/R and I/R + DMSO groups (p < 0.05) (Fig 8A). EAAT2 (glutamate transporter 1, GLT-1) is the major glutamate transporter expressed on astrocytes. GLT-1 is responsible to regulate glutamate concentration and ameliorate neurotoxicity in the brain by glutamate reuptake [12, 13]. The results of GLT-1 mRNA expression showed that I/R injury led to a reduction in the GLT-1 expression (p < 0.05), while, the GLT-1 expression level was backed to the control level following MK-4 administration in I/R group (Fig 8B).

### Reduction of pro-inflammatory cytokines induced by I/R injury following MK-4 administration in I/R rat model

**Fig 9:**
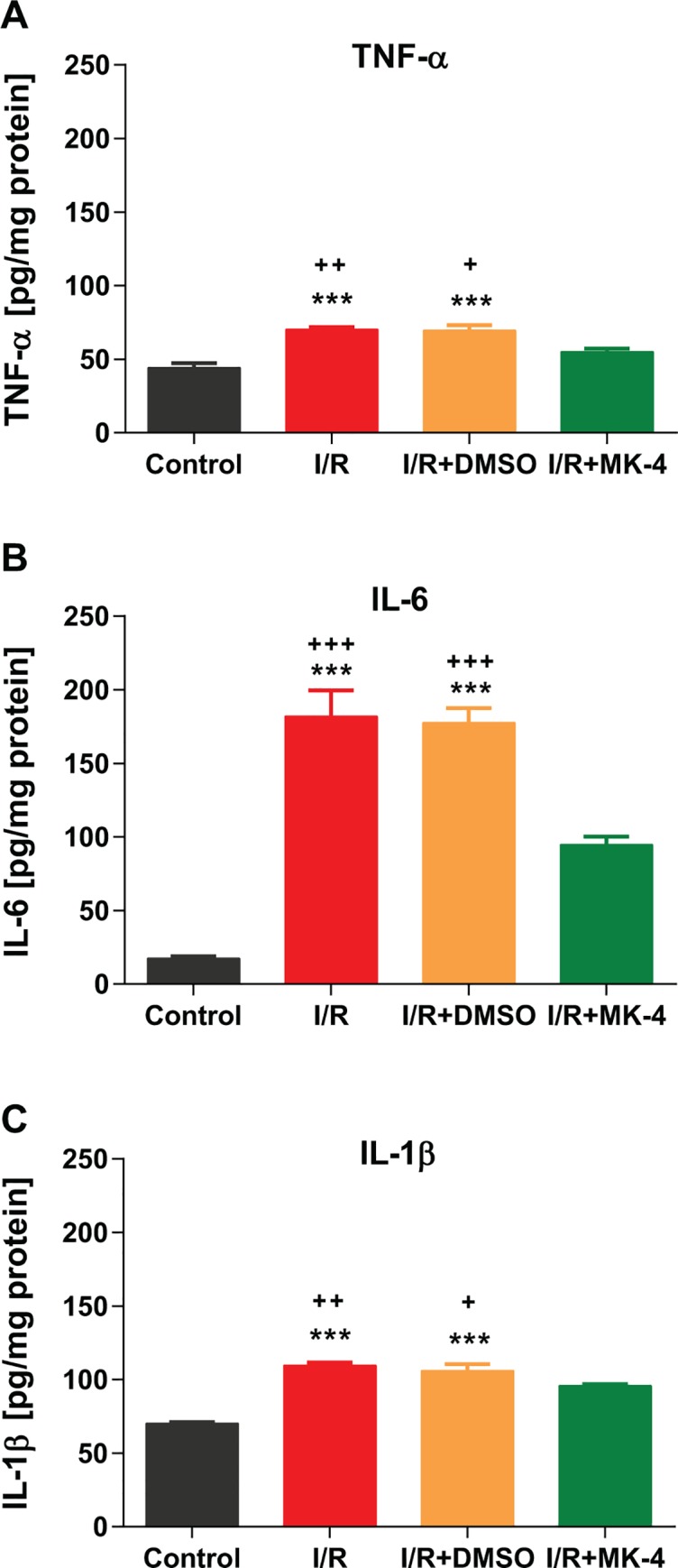
Inhibition of pro-inflammatory cytokines induced by I/R injury following MK-4 administration in I/R rat model. Pro-inflammatory cytokines level including **(A)** TNF-α, **(B)** IL-6 and **(C)** IL-1β were quantified in the hippocampus, 24 h after I/R injury. The level of TNF-α, IL-6 and IL-1β were increased following I/R injury, however, MK-4 application could prevent it. Data are presented as mean±SEM. *** p < 0.001 compared to control, + p < 0.05, ++ p < 0.01 and +++ p < 0.001 compared to I/R+MK4 group (n = 6).

The expression of pro-inflammatory cytokines as a predominant factor induced by I/R injury in the brain was detected using ELISA experiments in the hippocampus of different experimental groups. As shown in Fig 8, the level of TNF-α (p < 0.001, Fig 9A), IL-6 (p < 0.001, Fig 9B) and IL-1β (p < 0.001, Fig 9C) in the hippocampus of I/R and I/R + DMSO rats were significantly increased compared to the control group. While the amount of these pro-inflammatory cytokines was diminished after MK-4 injection (p < 0.05) compared to I/R and I/R + DMSO groups remarkably (Fig 9). These findings indicated that reperfusion following ischemic injury promotes the expression of pro-inflammatory factors and MK-4 application can suppress it.

## Discussion

Cerebral ischemic stroke is the most common type of stroke which can be induced by transient bilateral common carotid arterial occlusion in the animal model and mimics human cardiac arrest condition [7]. In this condition, blood flow to the brain is reduced and neurons are starved of oxygen and nutrients that quickly leads to cell death, especially in vulnerable regions of the brain such as the hippocampus [64]. In addition, following ischemia the damaged cells cannot function properly, due to compromised metabolism. There is also disruption of ion homeostasis, excessive release of excitatory neurotransmitters such as glutamate, calcium channel dysfunction, generation of oxidative stress and free radicals, activation of stress signaling, cell membrane disruption, inflammation which ultimately leading to necrotic and apoptotic cell death and elevation of total brain water content. Thus, ischemia can cause loss of structural and functional integrity of the brain. [72, 73]. It has been shown that following ischemia/reperfusion injury (I/R) various brain areas respond differently at distinct time-points [74]. Previous findings indicated that brain I/R injury led to cognitive function impairment [64]. Our findings here also showed that I/R injury causes hippocampal-dependent cognitive function impairment in both initial and reversal phases of Morris water maze and Y-maze test. These results remain consistent even for 7 days post I/R. Reduced number of surviving hippocampal neurons in different hippocampal subregions, oxidative stress, excitotoxity and induction of pro-inflammatory cytokines can be proposed as underlying mechanistic pathways for observed cognitive function deficits. In addition, glutamate receptors dysfunction can change calcium entrance and result in spatial learning and memory impairment [75, 76]. According to evidence, high oral doses of MK-4 in human (up to 135 mg) [77–79] and in animals (up to 200mg/kg) are effective, safe and have not toxic effects [37, 80, 81]. Our results showed that MK-4 at the dose of 100 mg/kg/day (similar to other studies [33–35]) has no significant effects on mortality rate in experimental group. Thus, we injected 400 mg/kg (200 mg/kg twice in a day i.p.) and observed reduction of mortality rate following cerebral I/R. Our investigation focuses on initial treatment I/R with high dose of MK-4. Certainly, next studies can clear other effects of this dosage on another disease in animals and human. Although Vissers and coworkers indicated that no significant associations were detected between dietary K1 and K2 vitamins intake with stroke risk [82]. But our data showed that administration of MK-4 immediately and 2 hours after I/R injury can compensate for the phenotypes induced by I/R. It was shown previously that MK-4 with activation of a Vitamin K-dependent protein—growth arrest-specific 6 (Gas6), present in hippocampal areas (CA1, CA3, and DG)—plays an important role for inhibiting Ca^2+^ influx [83, 84]. This inhibitive effect can be considered a potential explanation for how MK-4 application improves learning and memory disruption by I/R in behavioral evaluations. In addition to the cognitive function, MK-4 administration can diminish the anxiety-like behavior in I/R rat model. In the open field test the time spent in the center zone of the arena was considered as the anxiety index. The effects of transient global ischemia/reperfusion on open field performance are contradictory as there have been reports of increased anxiety or no changes in anxiety post-reperfusion [85, 86]. A study shows that increase mRNA expression and protein level of the nuclear mineralocorticoid receptor (MR) after cellular stress in rat hippocampus result in reduced anxiety behavior. This response, related to changes in gene expression is more likely to be manifest where protein synthesis is reduced for different periods after cerebral ischemia [87]. It could occur due to MK-4 enhancing neuronal survival, and may increase MR transcription and translation in these cells.

In addition to cognitive impairment, acute swelling after I/R occurs 15–30 min after reperfusion in this ischemia model [88]. Energy failure in I/R was caused to the imbalance of lactate, hydrogen, sodium and calcium ions. Also, oxidative stress induced by I/R increases accumulation of the vascular permeability factors such as hypoxia-inducible factor (HIF) and vascular endothelial growth factor (VEGF) which induce hyperpermeability by direct action on endothelial cells lead to increased capillary permeability and allowed permeation of certain proteins from vessels into the tissue to produced edema [89]. Changes of permeability were relatively frequent in the neocortex, thalamus, cerebellum, basal ganglia, hippocampus [90]. That is cleared that brain injury after I/R is associated with the inflammatory response, involving the infiltration and accumulation of immune cells and the activation of microglia and astrocytes [91]. Our results as well showed that 24h after transient global cerebral I/R, brain water content increased and it was reasonable to decrease oxidative stress and inflammatory factors by MK-4 exhibits a preventive action in BBB integrity and reduced total brain water content ultimately. However, MK-4 activate protein S that was found to significantly reduce total brain water content and improved and treated post-ischemia cerebral blood flow [83].

I/R injury in addition to increased total brain water content lead to neuronal cell death and apoptosis induction as the density of TUNEL positive cells and Bax/Bcl-2 mRNA expression ratio was increased. Bax is a pro-apoptotic protein that can damage the outer mitochondrial membrane and cause the release of Cytochrome C from mitochondria. Bcl-2 (an anti-apoptotic protein) is associated with the mitochondrial outer membrane and can inhibit the release of Cytochrome C. Furthermore, Bax can attach to Bcl-2 and stop its functions. It has been reported that dramatic downregulation of Bcl-2 and upregulation of Bax proteins in vulnerable tissues such as hippocampus at 24 h post-reperfusion [92]. The Bax/Bcl-2 ratio determines the capacity of cells to a death signal. On the opposite side, inhibiting the Bax expression can prevent protect cells against apoptosis [73]. Reports show that this ratio elevated in I/R and promote cell death [93]. Inflammatory molecules such as TNF-α increase apoptosis by enhancement Bax and Bax/Bcl-2 ratio. These molecules can also induce necrosis [94, 95]. As well as some of the studies mentioned that glucocorticoids activate their receptors and increase cell death by inflammation and excitotoxic injury after cerebral ischemia [96, 97]. Research indicates that Vitamin K2 reduces the pro-apoptotic proteins Bax in osteoblasts by increasing Gas6 protein [98]. Injection of MK-4 can potentially prevent the ROS generation [6] and calcium influx [83]. Moreover, the anti-inflammatory aspects of MK-4 decrease the Bax/Bcl2 ratio and block necrosis and apoptosis [99].

It was shown that primary cause of cellular macromolecule damage and apoptotic cell death after I/R is the overexpression of ROS, for instance, superoxide radicals. Increasing formation of ROS can deplete SOD after cerebral ischemic-reperfusion injury. It related to the high susceptibility of the hippocampus to oxidative damage. On the other hand, overexpression of SOD has been shown to reduce ischemic injuries [100]. Antioxidants compound targeted to reduce ROS and increase antioxidant enzymes that result in protection against ischemic injury. Moreover, there is evidence that overexpression of antioxidant enzymes can reduce oxidative stress in oxygen–glucose-deprived neurons and global cerebral ischemia [101, 102]. Although Vitamin K is not known as a classical antioxidant, research reports that Vitamin K1 and K2 (menaquinone-4) potently inhibit glutathione depletion-mediated oxidative cell death. Vitamin K1 and MK-4 or its metabolites indirectly blocked 12-lipoxygenase (12-LOX) enzymatic activity and prevented ROS generation significantly in developing oligodendrocytes challenged with arachidonic acid [6, 22]. The results of the present study as well demonstrate that the neuroprotective effect of treatment with MK-4 significantly elevated SOD levels in the hippocampus of the ischemic rat model and reduced oxidative stress. NO is synthesized from L-arginine to citrulline and acts as a regulator neuronal signaling. It can react with superoxide anion to produce peroxynitrite, an oxidative radical that causes protein nitration and lipid peroxidation [103]. NO• has stability in an environment with a lower oxygen concentration. Inducible synthase (iNOS) has been indicated as an important mediator of inflammatory responses during ischemia and reperfusion [104]. Furthermore, research shows that neurons produce NO by Ca^2+^ dependent activation of neuronal NOS (nNOS). This mechanism is related to the hyperactivation the N-methyl- D-aspartate (NMDA) receptor of glutamate that leads to cell death. Several stimuli, such as cytokine-mediated gene expression activation, strongly induce iNOS expression in astrocytes, allowing unregulated NO release by these cells; this may be damaging for the neighboring neurons after I/R in the hippocampus. Inflammatory factors such as bradykinin cause increasing NO production by induction of iNOS [105–107]. Our experiments show that administration of MK-4 can potentially reduce NO level in hippocampus tissue. It is likely that MK-4 reduced NO production by decreasing activity of neuroinflammation factors such as bradykinin [35], TNF-α [99], IL-β [108] and inhibiting calcium entrance [83].

Of primary importance are astrocytes which protect neurons in I/R injury. The roles of astrocytes are diverse: from the release of neurotrophic factors, control of fluid, ion and pH homeostasis, neurotransmitter scavenging to the management of metabolite and waste products [13]. Astrocyte morphology change significantly when they become reactive following I/R. Enhancement proliferation of reactive astrocytes to the injury site are associated with a sustained and progressive increase in GFAP levels in that cells. There is a close association with the degree of brain injury and over-expression of pro-inflammatory cytokines in I/R [109]. Chronic inhibition of glutamate transporters has been shown to significantly increase excitotoxic neuronal damage at the post-ischemic time. Reduced GLT-1 mRNA expression has been shown in the hippocampus following I/R [13]. Decreased releasing of inflammatory cytokines and iNOS coupled with regulated astrocytic response post-ischemia [13]. A study showed that vitamin K especially MK-4 regulate astrocyte function by contributing to electron transport and energy metabolism [110]. We could observe here that MK-4 increased GLT-1 mRNA in hippocampal tissue following I/R injury in rats. Also, MK-4 administration can markedly attenuate ischemia-induced GFAP gene transcription. It seems that MK-4 with anti-inflammatory actions through downregulation of astrocyte activation and preventing GLT-1 dysfunction can be a great candidate in investigations of novel therapeutic targets for I/R induced brain function deficits.

In general, inflammatory processes happen during the early phase of ischemic stroke and have a central role in the disease result. In addition to astrocytes, pro-inflammatory cytokines are released by different cell types such as leukocytes and microglia. These mediators modulate the reaction of many cell types in a number of diseases. Especially IL- 1β, IL-6 and TNF-α play a primary role in hippocampus inflammation responding to transient global ischemia/reperfusion [92, 111, 112]. Anti-inflammatory drugs can reduce suffering an ischemic stroke [112]. Vitamin K2 has been used as a therapeutic agent for the treatment of pain, inflammation and chronic diseases with an inflammatory background include cystic fibrosis, inflammatory bowel disease, pancreatitis, chronic kidney disease and osteoporosis [34–36]. Investigations about the anti-inflammatory activity of vitamin K1 and K2 is valuable, because of its very low toxicity. It has been suggested that vitamin K by inhibition of the cell signaling complex nuclear factor kappa-B (NF-κB) can induce a potent anti-inflammatory effect. IL-6 and TNF-α have a wide role in inflammatory diseases and many *in vivo* and *in vitro* studies revealed that Vitamin K catabolites at high levels of pharmacological doses have inhibitory effect on IL-6 and TNF-α release by suppressing NF-κB translocation to the nucleus in the brain [36, 83, 99]. Moreover, Vitamin K2 has an inhibitory effect on IL-1β function [108]. Our ELISA results showed that injection of 400 mg/kg MK-4 (200 mg/kg immediately and 2 h after I/R) can reduce the amount of IL-6, IL-1β and TNF-α in the hippocampus of I/R rat model. Reduction of pro-inflammatory cytokines production prevents brain damage and cell death.

Overall, the findings of the present study indicated the potential positive effects of MK-4 administration on ischemic injury associated with the reduction of total brain water content, nitric oxide anions production, GFAP mRNA expression, IL-6, IL-1β, TNF-α level, and Bax/Bcl2 mRNA expression ratio. On the other hand, vitamin K2 increased SOD activity and GLT-1 mRNA level which could inhibit oxidative stress, neurotoxicity, and cell death. Thus, the new findings of current research suggest a strong therapeutic potential of MK-4 against I/R brain injury that may be involved neuroprotection. Further future investigations for the detailed anti-inflammatory role of MK-4 and its impacts on protein levels of selected key candidate factors can potentiate this hypothesis.

## Supporting information

S1 Dataset The raw data of our findings for further meta-analysis. (XLSX)
